# Design of a CH_3_NH_3_PbI_3_/CsPbI_3_-based bilayer solar cell using device simulation

**DOI:** 10.1016/j.heliyon.2022.e09941

**Published:** 2022-07-14

**Authors:** Sidra Khatoon, Satish Kumar Yadav, Jyotsna Singh, Rajendra Bahadur Singh

**Affiliations:** a Department of Physics, University of Lucknow, Lucknow, Uttar Pradesh 226007, India; b Centre of Excellence in Renewable Energy Education and Research, Faculty of Science, University of Lucknow, Lucknow, Uttar Pradesh 226021, India

**Keywords:** Bilayer solar cell, Efficiency, Photovoltaic performance, Thickness

## Abstract

With lead-based light harvesters, perovskite solar cells (PSCs) have an efficiency of approximately 25.5%, making them a viable photovoltaic technology. The selection of the absorber materials for PSC in this work are (i) Cesium lead iodide (CsPbI_3_) with a 1.73eV bandgap as the first absorber layer, this halide imparts higher stability to perovskite solar cells (ii) CH_3_NH_3_PbI_3_ (MAPbI_3_) with a bandgap of 1.55eV is selected as the second absorber layer as this material provides better efficiency to the perovskite solar cells. SCAPS-1D simulation software is used to perform an efficiency analysis of perovskite-perovskite CsPbI_3_/MAPbI_3_ bilayer solar cell. For efficiency optimization of the perovskite-perovskite bilayer solar cell, we have tried to calibrate seven parameters of the cell. These parameters are (i & ii) selection of the electron and hole transport material (iii, iv & v) variation in the: defect density of bulk material, doping concentration and the thickness of absorber layers, (vi) variation in work function of front electrode (vii) varying interface defect density. After optimization, the efficiency (η) of bilayer PSC is estimated to be 33.54%. The other PV parameters observed in optimal efficiency condition are open-circuit voltage (V_OC_) = 1.34V, short-circuit current density (J_SC_) = 27.45 mA/cm^2^ and fill factor (FF) = 90.49%. The CsPbI_3_/MAPbI_3_ bilayer perovskite solar cell efficiency is roughly double the efficiency of single junction CsPbI_3_ or MAPbI_3_ PSC. Our analysis observed that the variation in the doping and defect density of narrow bandgap material profoundly impacts the efficiency of perovskite-perovskite bilayer solar cells compared to the wide bandgap material.

## 1 Introduction

Photovoltaic (PV) technologies have improved dramatically in efficiency over the years. First-generation solar cells, or crystalline technology, have achieved efficiencies up to 25% [1]. Second-generation solar cells or thin-film technology, have achieved efficiencies up to 29%, while third-generation solar cell technology, including dye-sensitised solar cells, perovskite solar cells (PSC), have attained efficiency up to 25.5% [2].

PSC is the whole new phase of photovoltaic because its efficiency increased sharply in a short duration of time i. e, from 3.8% to 25.5% in a decade. The reason for this accelerated progress can be assigned to some peculiar characteristics of perovskite material, such as tunable bandgaps (1.2eV–2.3eV), high absorption coefficients (more than 10^4^ cm^−1^), high mobility (up to 2000 cm^2^ V^−1^ s ^−1^), long diffusion lengths (more than 1000nm) and low exciton binding energies of (2–22 meV)charge carriers [3, 4]. However, the volatile constituents in hybrid organic-inorganic perovskite can cause thermal and chemical instability, which is a barrier to its commercialization [5]. Inorganic halide perovskites are made by replacing volatile organic components with cesium (Cs), and have recently gained a lot of attention due to their inherent inorganic stability and fair photovoltaic performance [6, 7]. The inorganic hybrid perovskite CsPbX_3_ (X = Cl, Br, I) has been found to have better thermal stability than the organic-inorganic halide perovskites [8, 9]. The cubic phase of cesium lead triiodide (CsPbI_3_) with a bandgap of 1.73 eV, is optimal for photovoltaic applications and at the same time it is also a perfect material for integrating tandem solar cells with lower bandgap solar cells [10, 11]. CsPbI_3_-based PSCs have improved their efficiency from 2.9 percent to 19.03 percent, with better stability, revealing that it has a huge potential for fabricating high-efficiency inorganic PSCs [12, 13].

In order to further enhance the efficiency of PSC, many groups are working on the fabrication of heterojunction of two similar structures, also called bilayer PSC. These bilayer formations enhance the solar spectrum absorption near the infra-red region, thereby producing higher current density. Duan et.al, worked on heterojunction of MAPbI_3_/CsSnI_3_ and achieved 21.64% efficiency [14]. Similarly, Ullah S et al.; demonstrated 15.89% efficiency in all-inorganic PSC with bilayer absorption scenario using device simulation [15]. Li et al. demonstrated 15.2% efficiency and better stability in quantum dots PSC through a bilayer absorption scenario [16].

Thus, PSC became a good choice in the field of solar energy, but due to the complex fabrication techniques of PSC, the invention of simulation tools in this field was promoted. Few solar cell simulation software are SCAPS-1D, AMPS-1D, Silvaco-TCAD, ASA. Using these simulation tools, various combinations of organic-inorganic perovskite materials can be tested, and optimal material combinations can be explored. Using SCAPS-1D recently Singh et.al [17], achieved 26.72% efficiency in (CH_3_NH_3_GeI_3_)/(FAMASnGeI_3_) configuration and Madan et. al [18], achieved 17.3% efficiency in Pb free (FACsPb_0.5_Sn_0.5_I_3_/Cs_2_AgBi_0.7_5Sb_0.25_Br_6_) configuration.

In this study, we have focused on optimizing the efficiency of perovskite-perovskite bilayer solar cell using SCAPS (a solar cell capacitance simulator) - a 1D simulation tool with CsPbI_3_ and MAPbI_3_ as the two absorber layers. The selection of MAPbI_3_ material is due to its higher efficiencies reported in the literature because of the favorable bandgap of 1.55eV, high mobility and long diffusion length of charge carriers. At the same time, the CsPbI_3_ material is chosen because it is thermally stable and CsPbI_3_ is also a potential partner for tandem devices because its bandgap (1.73eV) [19]. Hereby creating a heterojunction of CsPbI_3_/MAPbI_3_, which cannot be accomplished in conventional thin-film perovskite solar cells, we improve the efficiency of PSC from 14 percent to 33 percent. Up to our knowledge this is one of the highest recorded efficiencies for PSC till now. Our results reveal that the use of narrow-bandgap material MAPbI_3_ has increased the absorption range of solar spectra and the bilayer fabrication of solar cell have better aligned the energy levels, which promotes the carrier extraction, resulting in higher charge carrier generation and transport of carriers in comparison to single -CsPbI_3_ or MAPbI_3_ PSC.

The selection of Pb as one of the components in our work despite being toxic is addressed and resolved by Li et al. In their work they have fabricated DMDP (P, P′-di (2-ethylhexyl) [3, 4] methanediphosphonic acid) laminated ethylene vinyl acetate (EVA) tapes which can absorb Pb^2+^ ion. The solar cells are laminated using these tapes and 99.9% of Pb^2+^ ion absorption is reported [20].

## 2 Material and method

The fabrication of PSC is a complex process; hence, the scientific community primarily relies on simulation tools for material and process optimization. Various simulation tools like wx-AMPS, SCAPS-1D, Silvaco-TCAD, etc., are available online for research and design. Different research groups develop these simulation codes, and generally, their updates are available from time to time. We have selected the SCAPS (a solar cell capacitance simulator)-a 1D simulator for our simulation study developed by the University of Ghent's Department of Electronics and Information Systems (ELIS) [21]. The simulator solves the basic three differential equations, Poisson equation (equation 1), carrier continuity equation of electrons and holes (Eqs. (2) and (3)), to calculate the current-voltage characteristics, spectral response, and energy bands [22]. The equations used in the considered tool are as follows:

Poisson equation:

(1) $$\frac{d}{dx}\left(\varepsilon \left(x\right)\frac{d\psi }{dx}\right)=q\left[p\left(x\right)-n\left(x\right)+N_{D}^{+}\left(x\right)-N_{A}^{-}\left(x\right)+p_{t}\left(x\right)-n_{t}\left(x\right)\right]$$

Continuity equation for holes:

(2) $$\frac{1}{j}\frac{dJ_{P}}{dx}+R_{P}\left(x\right)-G\left(x\right)=0$$

Continuity equations for electrons:

(3) $$-\frac{1}{j}\frac{dJ_{n}}{dx}+R_{n}\left(x\right)-G\left(x\right)=0$$

Here, ε depicts the permittivity; the electrostatic potential is represented by ψ; q is the charge of an electron, the doping concentrations of donor and acceptor ions are N_D_^+^ and N_A_^−^, respectively; the electron and hole current densities are J_n_ and J_p_, respectively. G(x) is the electron and hole generation rate while the electron and hole recombination rates are R_n_(x) and R_p_(x), respectively; and n and p are free electrons, and hole concentration, n_t_ and p_t_ are concentrations of trapped electrons and holes, respectively.

In our simulation study, two different absorber layers are selected, and the selection of the absorber material is based on (i) the methylammonium lead iodide (MAPbI_3_) for higher efficiency while (ii) caesium lead iodide (CsPbI_3_) for better stability. The present simulation is performed in three steps.

**Figure 1:**
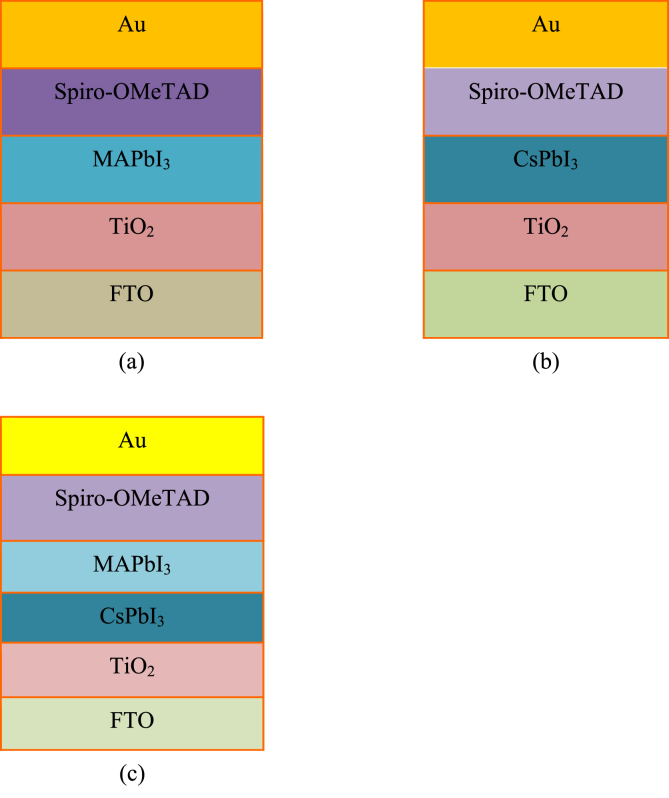
Simulated structure of (a) MAPbI_3_ based PSC (b) CsPbI_3_ based PSC (c) Bilayer structure of PSC.

In the first step, we have simulated the performance of a single-junction MAPbI_3_ PSC. The device configuration of MAPbI_3_ single-junction PSC is FTO/TiO_2_/MAPbI_3_/Spiro-OMeTAD/Au, as presented in Figure 1(a).

In the second step, another single-junction CsPbI_3_ based PSC is simulated. The configuration of the CsPbI_3_ single-junction selected is FTO/TiO_2_/CsPbI_3_/Spiro-OMeTAD/Au, as presented in Figure 1(b).

We moved to the final bilayer PSC simulation after comparing the simulated findings to the experimental data.

The perovskite-perovskite bilayer solar cell with the two absorber layers CsPbI_3_ and MAPbI_3_ is simulated in the third step. It is further enhanced by adjusting the thickness, defect density in the bulk and at the CsPbI_3_/MAPbI_3_ interface, N_A_^-^of the two absorber layers, the work function of the front contact and alternative materials for the Electron Transport Layer (ETL) and Hole Transport Layer (HTL). Its configuration is FTO/TiO_2_/CsPbI_3_/MAPbI_3_/Spiro-OMeTAD/Au which is depicted in Figure 1(c). The description of different layers in PSC are as follows: (i) Fluorine Doped Tin Oxide (FTO) acted as a front electrode (ii) TiO_2_ is used as Electron Transport Layer (ETL) (iii) CsPbI_3_ as the first absorber layer (iv) MAPbI_3_ as second absorber layer (v) Spiro-OMeTAD as Hole Transport Layer (HTL) (vi) Au as a back electrode.

Table 1 illustrates the material properties of each layer of the PSC as taken from the various published work. In the Table, N_C_ and N_V_ stand for effective conduction and valence band density, E_g_ for bandgap, N_A_ and N_D_ for acceptor and donor density, μ_p_ & μ_n_ for hole & electron mobility, N_t_ for defect density, ε_r_ for relative permittivity, χ for electron affinity.

**Table 1:** Material properties of the absorber layer, ETL and HTL.

Parameters	FTO	TiO_2_	CsPbI_3_	MAPbI_3_	Spiro-OMeTAD
Thickness/nm	500	50	100–1000	100–1000	200
*E*_g_/eV	3.5	3.2	1.73	1.5	2.8
*χ*/eV	4.0	4	3.95	3.9	2.05
*ε*_r_	9.0	9.0	10	30	3.0
*N*_c_/cm^−3^	2.2×10^18^	1.0×10^21^	2×10^18^	2.5×10^20^	2.2×10^18^
*N*_v_/cm^−3^	1.8×10^19^	2.0×10^20^	5×10^18^	2.5×10^20^	2.5×10^19^
*μ*_n_/(cm^2^/V·s)	20	20	20	50	2.0 × 10^−4^
*μ*_p_/(cm^2^/V·s)	10	10	20	50	2.0 × 10^−4^
*N*_D_/cm^−3^	1×10^19^	-	0	0	0
*N*_A_/cm^−3^	0	1.0×10^18^	1.0×10^15^	1.0×10^18^	2.0×10^19^
*N*_t,bulk_/cm^−3^	-	1×10^15^	2.07×10^14^	1×10^15^	1×10^15^
References		[23, 24]	[27, 28, 25, 26]	[29]	[30, 31]

The light spectrum of AM 1.5G falls on the front electrode and, after absorption of a shorter wavelength, transmits to the bottom absorber layer for higher energy photon absorption. The simulation is performed at 300K temperature, the electron and hole velocities are taken as 10^7^ cm/s. The front contact of PSC has a 4.4eV work function, whereas the back contact has a 5.1eV work function. For CsPbI_3_ based solar cells, the interface parameters include a neutral defect type with a characteristic energy of 0.1eV and located at mid-gap. The captured cross-section for both the electron and hole is 1 × 10^−15^ cm^2^. In the bilayer PSC, the interface parameters at the junction of two absorber layer (CsPbI_3,_/MAPbI_3_) are interface defect density 1× 10^10^ cm^−3^, neutral defect type with a characteristic energy of 0.1eV and located at mid-gap. The captured cross-section for both the electron and hole is 1 × 10^−19^ cm^2^.

## 3 Results

**Figure 2:**
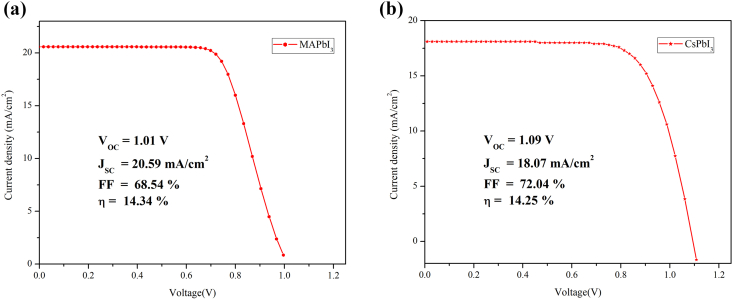
J-V curve of single-junction (a) MAPbI_3_ and (b) CsPbI_3_ based PSC.

In the present study, initially, single-junction solar cells CsPbI_3_ and MAPbI_3_ were simulated using SCAPS-1D software. The MAPbI_3_ based solar cell obtained Photovoltaic Conversion Efficiency (PCE) = 14.34% Fill Factor (FF) = 68.54%, short-circuit current density (J_sc_) = 20.59 mA/cm^2^, open-circuit voltage (V_oc_) = 1.01V as shown in Figure 2 (a), while CsPbI_3_ based device produces PCE = 14.25%, FF = 71.98%, V_oc_ = 1.09V, J_sc_ = 18.06 mA/cm^2^ as shown in Figure 2 (b). The V_oc_ of CsPbI_3_ PSC is higher than the MAPbI_3_ PSC because of the higher bandgap of the latter, while J_sc_ of MAPbI_3_ is higher CsPbI_3_ because of better absorption of the solar spectrum by MAPbI_3_ as compared to CsPbI_3_ PSC.

Tables 2 and 3 reveal that the simulated and experimental results of both CsPbI_3_ and MAPbI_3_ PSC closely match each other, validating our simulation study [24, 26].

**Table 2:** Experimental and simulated parameters of single-junction MAPbI_3_ PSC.

	V_oc_ (V)	J_sc_ (mA/cm^2^)	FF (%)	η (%)
Experimental [32]	1.097	18	74.1	14.67
Simulated	1.01	20.59	68.54	14.34

**Table 3:** Experimental and simulated parameters of single-junction CsPbI_3_ PSC.

	V_oc_(V)	J_sc_ (mA/cm^2^)	FF (%)	η (%)
Experimental [30]	1.06	21.85	64.0	14.83
Simulated	1.09	18.07	72.04	14.25

The study was further continued on all perovskite bilayer solar cell. The two absorber layers in all perovskite bilayer solar cell are MAPbI_3_ and CsPbI_3._ The heterojunction mentioned above allows near-infra-red absorption and hence permits broader absorption of the solar spectrum. It also helps in increasing photo-generation of charge carriers and therefore producing higher current density. The obtained PCE from bilayer PSC = 20.39%, FF = 79.21%, J_SC_ = 27.38 mA/cm^2^, V_OC_ = 0.93V. The optimization of the bilayer PSC is further obtained by varying the material of ETL and HTL. At the same time, we have varied the thickness, N_t_, and N_A_ of the absorber layers. Furthermore, variation of electrode work functions for front contacts of the PSC was observed to achieve optimal efficiency.

### 3.1 Effect of the ETL

**Figure 3:**
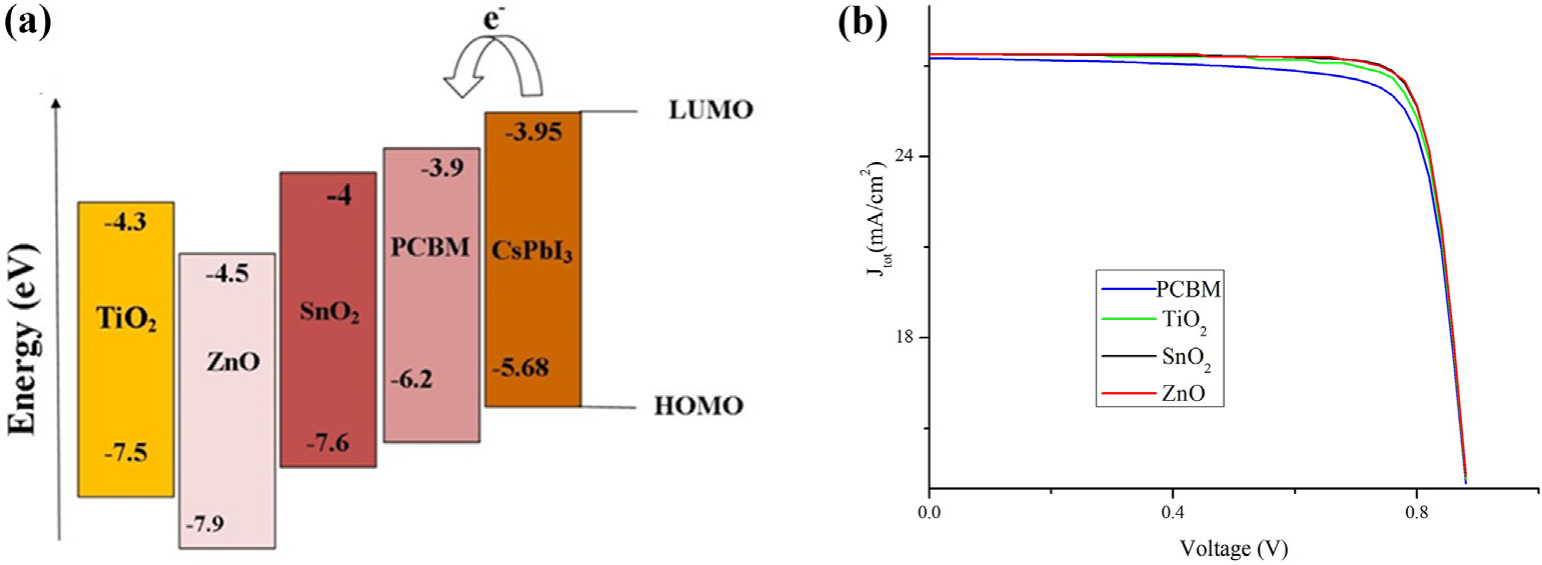
(a): Band alignment between CsPbI_3_ with respect to different ETLs. (b) J-V curve of various ETL on perovskite-perovskite PSC.

In a device architecture, electron transport layers must meet certain requirements, including (i) high band-gap to allow effective light-collection, (ii) well-matched alignment of energy-level for efficient electron transfer and preventing holes, and (iii) high value of electron mobility to reduce accumulation of charge-carriers [33, 34, 35]. This portion of the study will explore the impact of various ETL materials on PSC PV performance by studying the alignment of energy levels. Table 4 shows the value of the different parameters of various ETL materials, which are collected from various published works. The J-V characteristic curve of PSC with all ETL materials considered in Table 4 is shown in Figure 3(b). From Table 5, we observe that ZnO, TiO_2_, SnO_2_ presents comparable efficiency, i.e., 20.65%, 20.39%, 20.64%, respectively. The justification for the highest efficiency of ZnO can be attributed to better alignment of energy band between the conduction band and lowest unoccupied energy level (LUMO) of CsPbI_3_ and high mobility of charge carriers, as shown in Figure 3(a). At the same time, we observe from Table 5 that the PCBM ETLs show the lowest J_sc_, FF, and efficiency compared to other ETL materials. This observation can be attributed to the low bandgap and low mobility of charge carriers in PCBM, and hence PCBM is not suitable for the bilayer PSC device. At the same time, slight misalignment in the energy level between the PCBM and the CsPbI_3_ deteriorates the performance of PSC. From Table 5, it can be seen ZnO shows the best performance in comparison to other ETLs, and therefore it is the best ETL for the CsPbI_3_ absorber layer. Therefore, it is evident from our simulation study that the inorganic materials perform better than the organic materials.

**Table 4:** Material properties of various ETLs used in the simulation.

Parameters	TiO_2_	ZnO	SnO_2_	PCBM
Thickness/nm	50	50	50	50
*E*_g_/eV	3.2	3.3	3.5	2.0
*χ*/eV	4	4.0	4.0	3.9
*ε*_r_	9.0	9	9	3.9
*N*_c_/cm^−3^	1.0×10^21^	2.2×10^18^	4.36×10^18^	2.5×10^21^
*N*_v_/cm^−3^	2.0×10^20^	1.8×10^19^	2.52×10^19^	2.6×10^21^
*μ*_n_/(cm^2^/V·s)	20	100	20	0.2
*μ*_p_/(cm^2^/V·s)	10	25	10	0.2
*N*_D_/cm^−3^	1.0×10^18^	1.0×10^18^	1.0×10^18^	2.93×10^17^
*N*_.A._/cm^−3^	-	-	-	-
*N*_t,bulk_/cm^−3^	1×10^15^	1.0×10^15^	1×10^15^	1×10^15^
References	[23, 24]	[24]	[13, 24]	[24]

**Table 5:** PV performance with different ETL materials.

PSC structure	V_oc_ (V)	J_sc_ (mA/cm^2^)	FF (%)	η (%)
FTO/TiO_2_/MAPbI_3_/CsPbI_3_/Spiro	0.94	27.38	79.21	20.39
FTO/SnO_2_/MAPbI_3_/CsPbI_3_/Spiro	0.93	27.39	80.21	20.64
FTO/ZnO/MAPbI_3_/CsPbI_3_/Spiro	0.93	27.39	80.28	20.65
FTO/PCBM/MAPbI_3_/CsPbI_3_/Spiro	0.94	27.25	77.73	19.95

### 3.2 Effect of the HTL

**Figure 4:**
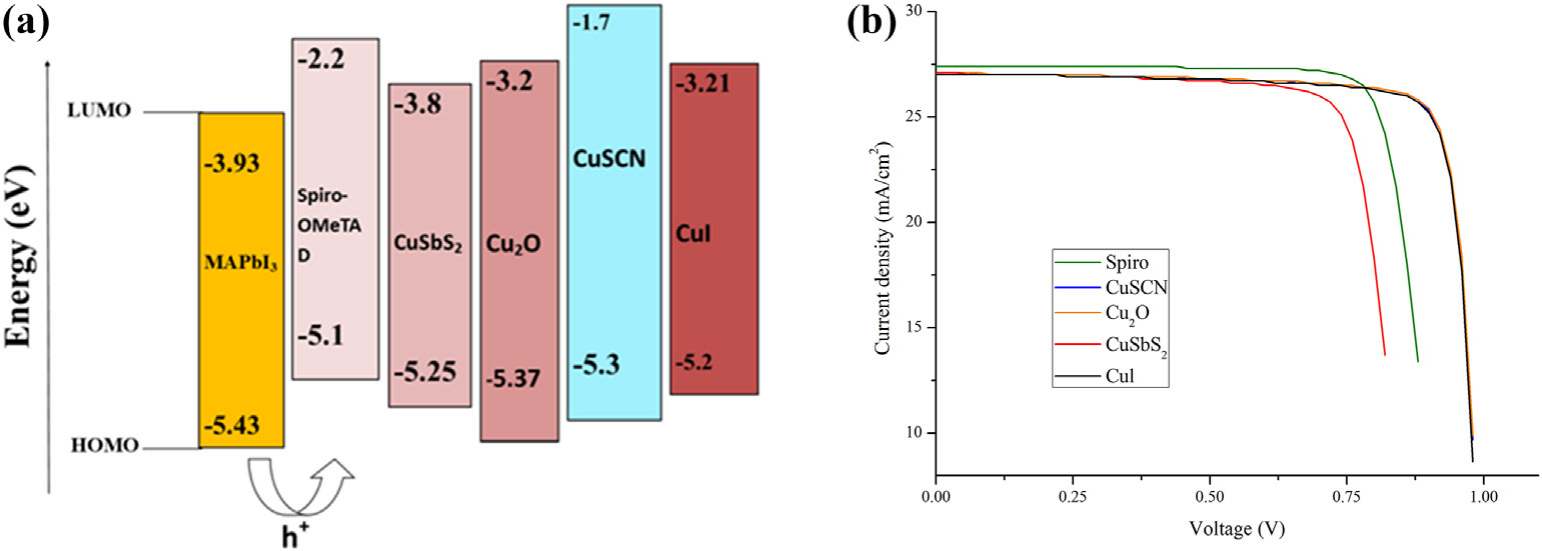
(a): Band alignment between CsPbI_3_ with respect to different HTLs. (b) J-V curve of PSC with different HTL.

The HTL must have adequate energy levels to provide the necessary driving force for charge transfer (i.e., the highest occupied molecular orbital, HOMO, energy levels of the selected hole transport material (HTM) must be slightly superior to that of the perovskite materials). It should have a high hole transfer efficiency to enhance hole conduction and prevent charge recombination (potentially >10^−3^ cm^2^V^−1^ s^−1^). The unstable, expensive (dopants, additives) organic HTMs (spiro-OMeTAD, PEDOT: PSS, PTAA, and P3HT) leads to the incorporation of inorganic HTMs (CuI, Cu2O, CuO) with high mobility and carbon-based HTMs for stability [31]. This section discusses the impact of the absorber and various organic and inorganic HTL material interfaces with ZnO as ETL material on the performance of the PSC. Figure 4(a) depicts the energy level alignment of the various HTL material with respect to the MAPbI_3_ as an absorber layer. Table 6 shows the values of different parameters of various HTMs, and Table 7 illustrates the values of V_OC_, J_SC_, FF and η of the resulting PSC. From Table 7 we gather that CuSCN shows the best performance (PCE = 22.81%, V_oc_ = 0.99V, J_sc_ = 27.07 mA/cm^2^, and FF = 84.9%) with respect to other HTL materials simulated. This behavior is attributed to better alignment of the energy level of the valence band of the absorber layer and the highest occupied molecular orbital (HOMO) of the CuSCN. After CuSCN, the performance of PSC in decreasing order is Cu_2_O, CuI, CuSbS_2_. The performance parameters with minimal suitable HTL (CuSbS_2_) are PCE = 18.57%, V_oc_ = 0.88V, J_sc_ = 27.07 mA/cm^2^, and FF = 77.92%. Among all the HTMs simulated in our study, the CuSCN showed the best performance, followed by CuI. The reason is that the deeper energy level alignment contributes to the higher V_oc,_ and also, the bandgap of CuSCN (3.4eV) is high enough to block the electron transport completely. At the same time, it effectively transports the holes to the back electrode. Also, its high optical transparency in the 300 nm–900 nm wavelength range allows better light absorption in the absorber layer and, therefore, contributes to the higher J_sc_. Thus the simulation reveals that inorganic HTMs perform better than the organic HTMs.

**Table 6:** Material properties of various HTLs used in the simulation.

Parameters	Cu_2_O	CuSCN	CuI	CuSbS_2_	Spiro-OMeTAD
Thickness/nm	150	150	150	150	150
*E*_g_/eV	2.17	3.4	3.1	1.58	3.0
*χ*/eV	3.2	1.9	2.1	4.2	2.45
*ε*_r_	7.11	10	6.5	14.6	3.0
*N*_c_/cm^−3^	2.02×10^17^	1.7×10^19^	2.8×10^19^	2.0×10^18^	2.2×10^18^
*N*_v_/cm^−3^	1.1×10^19^	1.8×10^19^	1.0×10^19^	2.0×10^19^	1.8×10^19^
*μ*_n_/(cm^2^/V·s)	200	2.0 × 10^−4^	100	49	2.0 × 10^−4^
*μ*_p_/(cm^2^/V·s)	80	2.0 × 10^−1^	43.9	49	2.0 × 10^−4^
*N*_D_/cm^−3^	-	-	-	-	-
*N*_A_/cm^−3^	1.0×10^18^	1.0×10^18^	1.0×10^18^	1.0×10^18^	2.0×10^18^
*N*_t,bulk_/cm^−3^	1×10^15^	1.0×10^14^	1×10^15^	1×10^15^	1×10^15^
References	[36]	[24]	[36, 37]	[29]	[30, 31]

**Table 7:** PV performance with different HTL materials.

PSC structure	V_oc_ (V)	J_sc_ (mA/cm^2^)	FF (%)	η (%)
FTO/ZnO/MAPbI_3_/CsPbI_3_/Spiro	0.93	27.39	80.28	20.65
FTO/ZnO/MAPbI_3_/CsPbI_3_/CuSCN	0.99	27.07	84.9	22.81
FTO/ZnO/MAPbI_3_/CsPbI_3_/Cu_2_O	0.99	27.07	84.93	22.83
FTO/ZnO/MAPbI_3_/CsPbI_3_/CuSbS_2_	0.88	27.07	77.92	18.57
FTO/ZnO/MAPbI_3_/CsPbI_3_/CuI	0.99	27.04	84.71	22.69

### 3.3 Effect of absorber layer thickness

In bilayer PSC different portion of the solar spectrum is absorbed by separate absorber layer, the top layer absorbs shorter wavelength while the bottom absorber layer imbibes longer wavelength photons. The thickness of absorber layers significantly affects the V_OC_, J_SC_, FF and η of PSCs. In this section, we have analyzed the optimal thickness of absorber layers in PSC and this is achieved by analyzing the properties of PSC on variation of the thickness of absorber material. At first, the thickness of the top layer was changed from 100nm to 1000nm keeping the MAPbI_3_ layer thickness fixed and vice-versa. In this way, the optimum thickness was estimated, which was selected for the top absorber layer.

**Figure 5:**
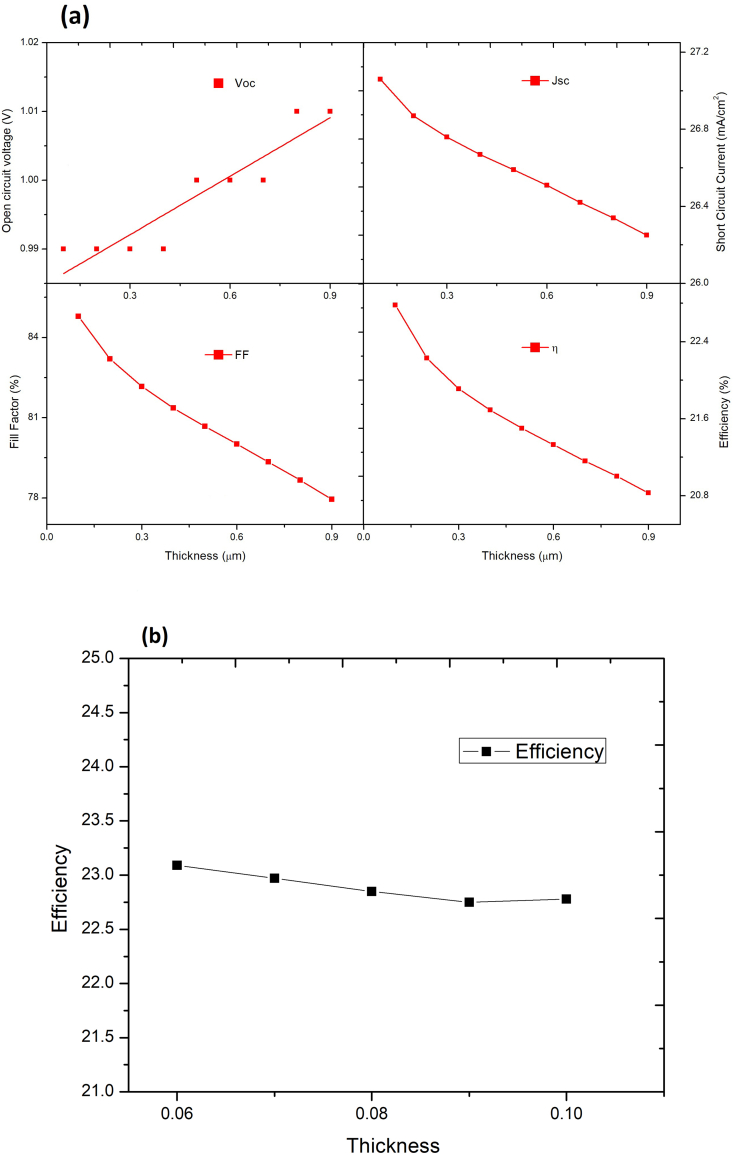
(a): Effect of CsPbI_3_ thickness variation keeping MAPbI_3_ thickness fixed on PV performance, (b): Variation in efficiency with thickness below 100nm.

**Figure 6:**
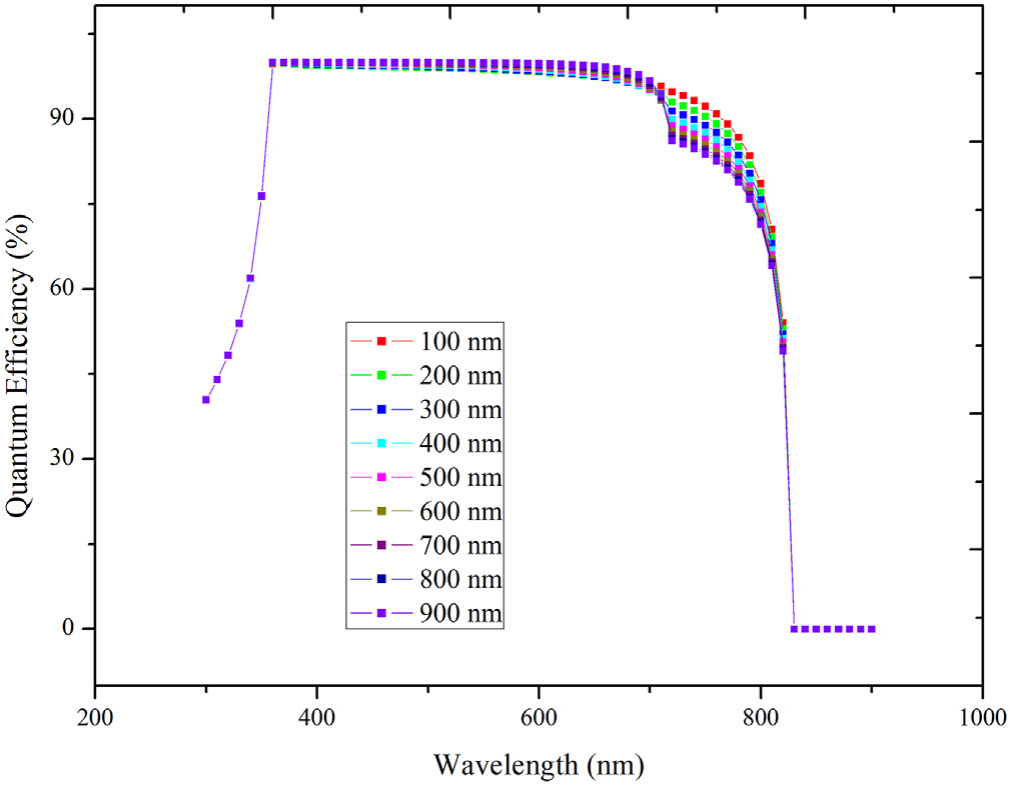
Quantum efficiency with variable thickness of CsPbI_3_ keeping MAPbI_3_ thickness constant.

In Figure 5(a) we have analyzed the properties of PSC with the change in thickness of CsPbI_3_ absorber layer (100nm–1000nm). We observe that there is a slight reduction in J_sc_ and a slight increment in V_OC_ on changing the thickness. For efficiency of the cell from Figure 5(b) we observe that the efficiency of the resulting PSC starts decreasing as the thickness of the CsPbI_3_ absorber layer increases. Figure 6 shows the effect of thickness variation in CsPbI_3_ on the quantum efficiency of the PSC. The maximum efficiency is achieved at 100 nm thickness, and therefore the thickness of CsPbI_3_ is fixed at 100nm in the rest of the studies. There is a negligible rise in efficiency less than (0.05%/10nm) below 100nm, as shown in Figure 5(b). Also, it is difficult to fabricate such a thin layer. Therefore, the optimal thickness of CsPbI_3_ is fixed at 100nm elsewhere in the study.

**Figure 7:**
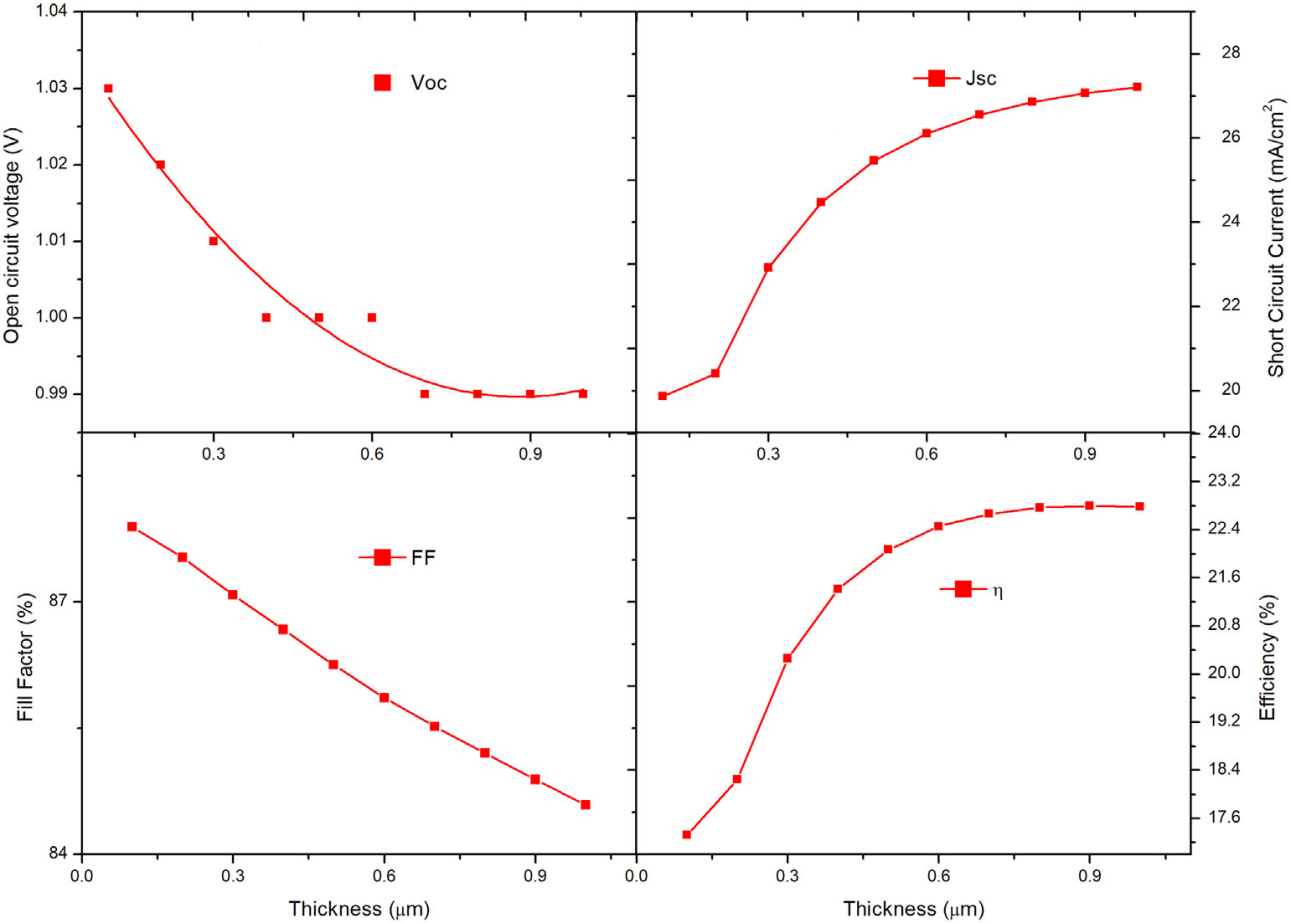
Effect of MAPbI_3_ thickness variation keeping CsPbI_3_ thickness fixed on PV performance.

**Figure 8:**
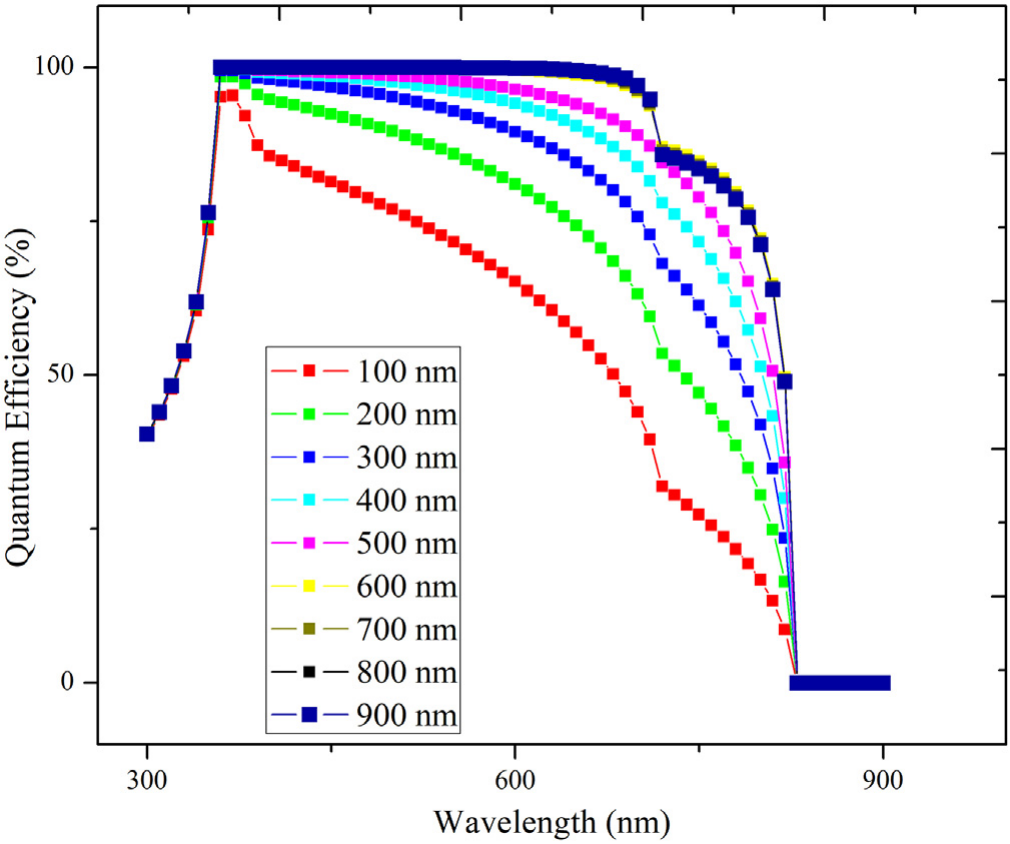
Quantum efficiency with variable thickness of MAPbI_3,_ keeping CsPbI_3_ thickness constant.

Now the thickness of CsPbI_3_ was fixed at 100nm while that of MAPbI_3_ was varied from 100nm to 1000nm. As observed from Figure 7, the J_sc_ is significantly improved from 20 mA/cm^2^ to 27 mA/cm^2^ due to increased light absorption, which induces a higher photo-generation of charge carriers. The V_oc_ reduces from 1.02V to 0.99V with increased thickness because of an increase in reverse saturation current (J_o_), increasing carrier recombination. The FF declines from 87 to 84% due to the rise in the series resistance (R_s_) of the PSC. Figure 8 shows the effect of thickness variation in MAPbI_3_ on the quantum efficiency of the bilayer PSC. The highest PCE and quantum efficiency is attained at 900nm. Therefore, the MAPbI_3_ thickness is fixed at 900nm elsewhere in the study. Our study shows that bilayer PSC performance is more dependent on narrow bandgap material thickness than on wide bandgap material thickness. The bandgap of the CsPbI_3_ absorber layer is 1.73 eV, hence it can absorb wavelengths up-to 650–700nm, whereas MAPbI_3_ absorber layer bandgap is of the order of 1.55eV hence it is capable of absorbing wavelengths up-to 900nm. Therefore, increase in the thickness of the CsPbI_3_ absorber layer creates hindrance to higher wavelength photons to reach the MAPbI_3_ absorber layer which results in the efficiency decrease of the resulting solar cell.

### 3.4 Effect of variation in defect density

The low-temperature and simple processing in perovskite halides result in significant defects at the grain boundary and interface. The presence of these defects reduces crystal quality. Defects cause perovskites to become active and prone to degradation, resulting in non-radiative recombination, impacting device performance and stability [38]. The defect density (N_t_) of absorber layers is tuned to see its effects on perovskite-perovskite bilayer PSC's performance. The SRH model examined the impact of (N_t_) in both absorber layers of PSC.

(4) $$R_{SRH}=\frac{np-n_{i}^{2}}{\tau_{p}\left(n+n_{i}\right)+\tau_{n}\left(p+p_{i}\right)}$$

(5) $$\tau_{n,p}=\frac{1}{\text{σ}{\text{ν}}_{th,n,p^{\text{γ}}}N_{t}}$$

Where,

σ: capture cross-sectional area

n: electron concentration

p: hole concentration

τ_p_: electron lifetime.

τ_n:_ hole lifetime.

**Figure 9:**
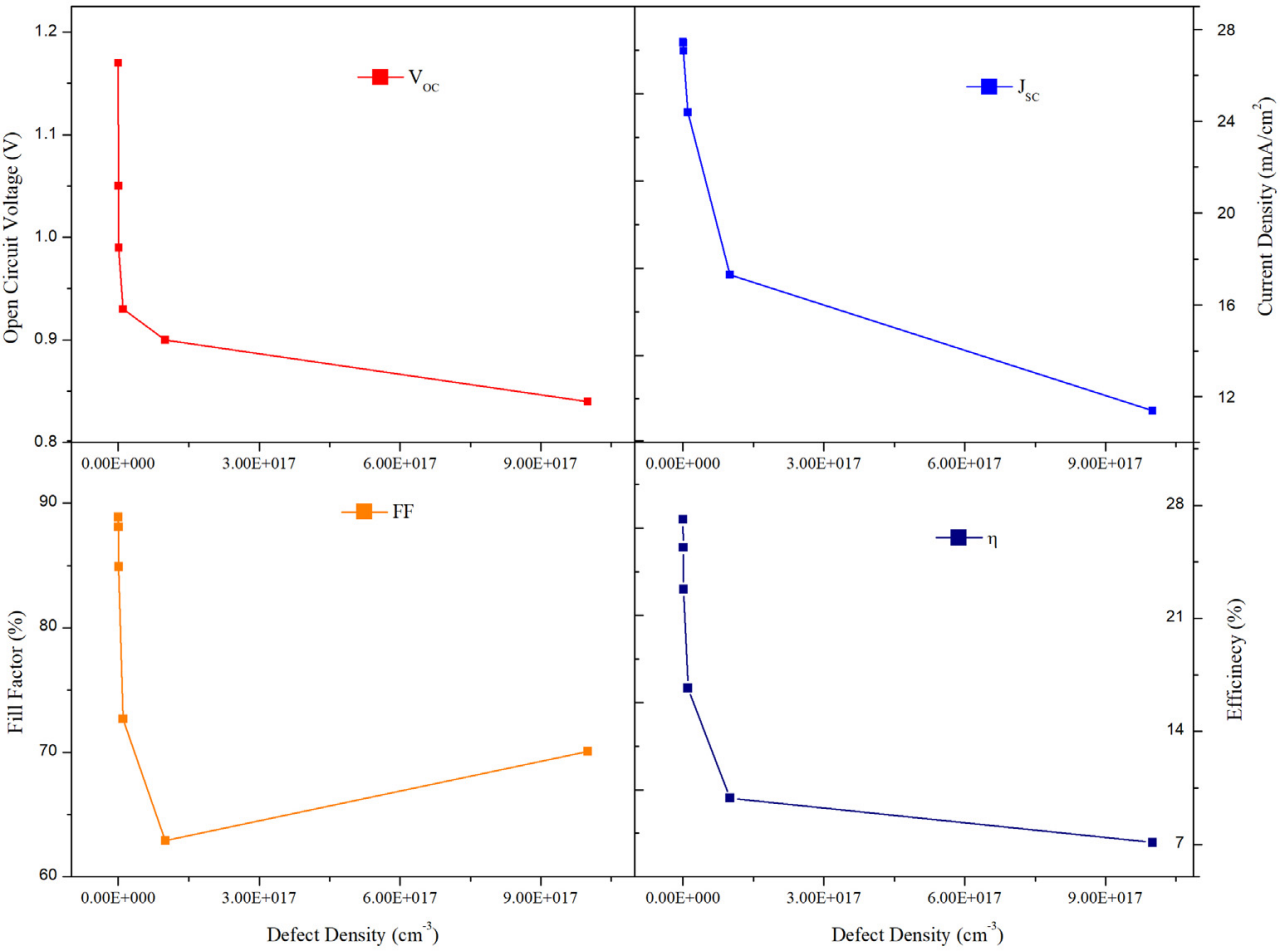
Effect of N_t_ variation in MAPbI_3_ keeping the N_t_ of CsPbI_3_ fixed on PV performance.

Eq. (4) shows that shokeley read hall recombination rate R_SRH_ which is inversely proportional to lifetime of charge carriers while Eq. (5) which shows the dependence of charge carrier lifetime on the defect density of the material. These equations imply that shockley read hall recombination rate R_SRH_ is directly proportional to the defect density of material. Thus, on increasing defect density the shokeley read hall recombination rate R_SRH_ increases by suppressing the current density and hence reducing the efficiency of solar cell. The above equation represents the maximum and minimum value of life time of charge carriers and defect density. These equations will give rough idea of life time and defect density but in real simulation more advanced algorithms are used for the estimation of these values at each layer on both sides of layer. The N_t_ of the MAPbI_3_ was changed between 1 ×10^13^ cm^−3^ to 1× 10^18^ cm^−3^, while the N_t_ of the CsPbI_3_ was kept constant at 1×10^14^ cm^−3^. As the N_t_ of the MAPbI_3_ absorber layer increases, efficiency falls from 27.13% to 7.13% the J_SC_ drops from 27.45 mA/cm^2^ to 11.38 mA/cm^2^ and the V_OC_ drops from 1.11 V to 0.84 V and FF was reduced from 88.88% to 70.09% and as shown in Figure 9. Therefore, from Figure 9, we conclude that 1× 10^13^ cm^−3^ to 1× 10^15^ cm^−3^ is the optimal range as the variation in V_oc_, Jsc, FF and η are negligible, but after 1×10^15^ cm^−3,^ the efficiency falls sharply.

**Figure 10:**
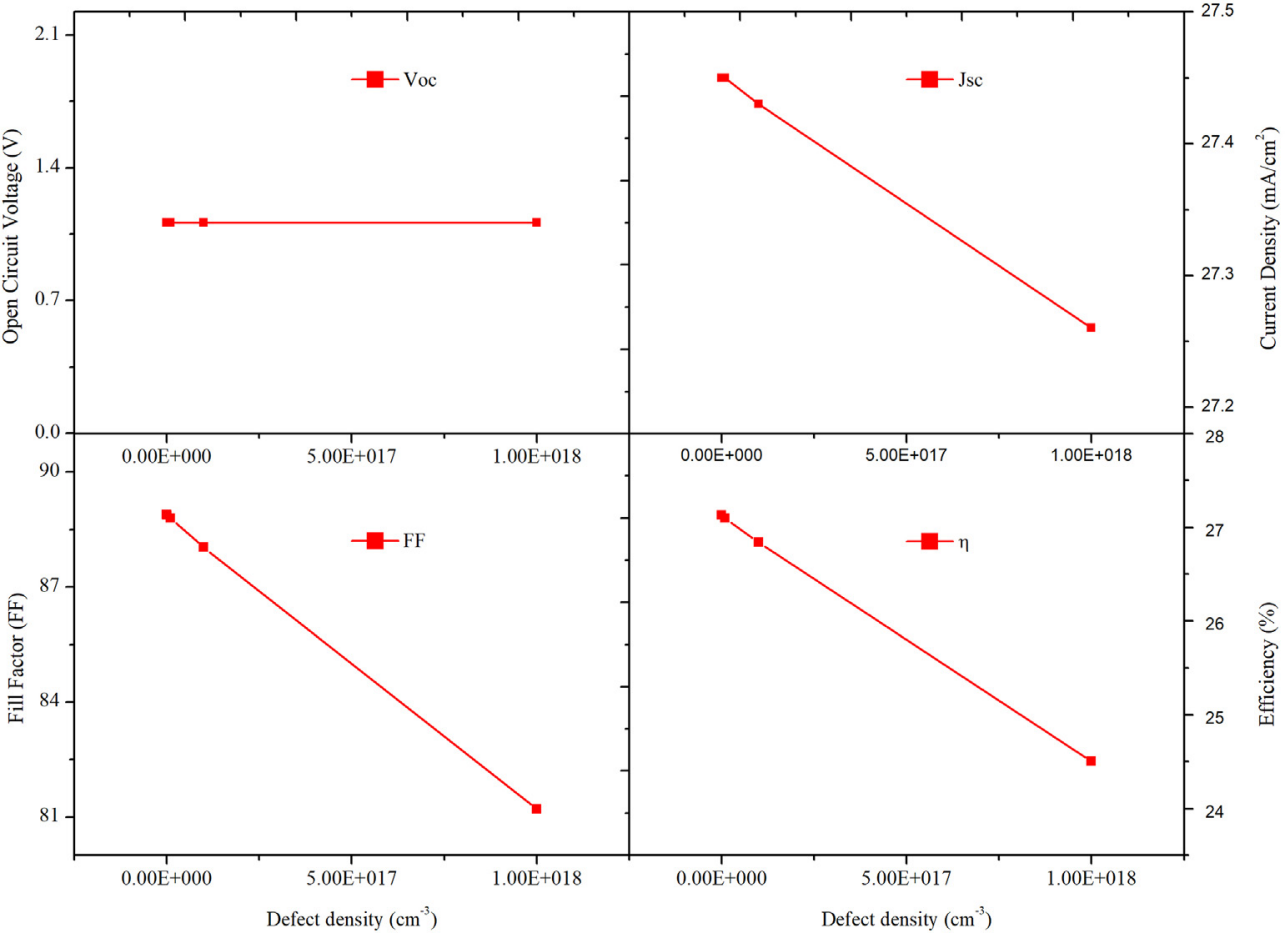
Effect of N_t_ variation in CsPbI_3_ keeping the N_t_ of MAPbI_3_ fixed on PV performance.

Similarly, the N_t_ of the CsPbI_3_ absorber layer is varied, while the N_t_ of MAPbI_3_ absorber layer is fixed at 1 ×10^13^ cm^−3^. It can be seen from Figure 10, the V_OC_ and J_sc_ do not diminish significantly as the N_t_ of CsPbI_3_ rises from 1 × 10^13^ cm ^−3^ to 1× 10^18^ cm^−3^, but FF drops from 88.88 to 81.21%, and the PCE drops from 27.19 to 24.50%. It was determined that when the N_t_ increases, more recombination centers are created, enhancing carrier recombination within the absorber layer while also reducing carrier lifetime, lowering the solar cell's device performance [39, 40]. As the number of recombination centers increases, the shunt resistance decreases, lowering the device's V_OC,_ as evident from Eqs. (6) and (7). According to the present research findings, when the N_t_ is 1× 10^13^ cm^−3^ for both absorber layers, the PV characteristics improves to, current density is 27.45 mA/cm^2^,V_OC_ is 1.01 V FF 88.88%, and efficiency 27.13%. Thus, the analysis reveals that the N_t_ of the absorber material has a significant impact on device performance. Also, bilayer.

PSC performance is more dependent on narrow bandgap material defect density than on wide bandgap material density. Various defect passivation techniques are reported in the literature such as incorporating different additives in perovskite absorber layer using different deposition techniques. Annie et.al, reported hybrid chemical vapor deposition technique and zhu et.al, reported addition of ethylamine alcohol for defect passivation [41, 42]. The value of N_t_ for the MAPbI_3_ is 1 ×10^15^ cm^−3^ and for CsPbI_3_ it is 2.07×10^14^ cm^−3^ is used elsewhere in the study.

(6) $$FF=FF_{\text{o}}\left(1-\frac{1}{r_{sh}}\right)$$

(7) $$FF=\frac{\nu_{oc}-ln\left(\nu_{oc}+0.72\right)}{\nu_{oc}+1}$$

Where,

νoc=Voc/(kTq)

FF_o_: ideal FF in the absence of any parasitic resistance

$r_{sh}:\text{shuntresistance}$

${\text{V}}_{\text{oc}}:\text{opencircuitvoltage}$

k: Boltzmann constant.

T: Temperature

q: elementary charge.

### 3.5 Effect of doping concentration

**Figure 11:**
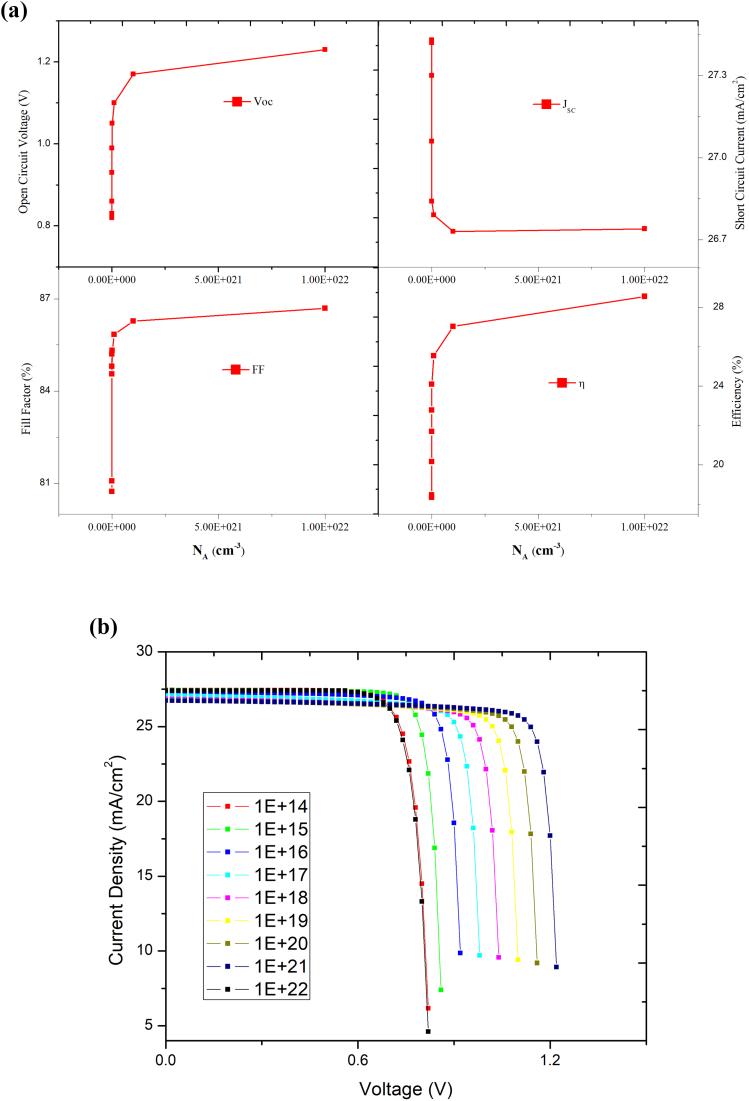
Effect of N_A_ variation in MAPbI_3_ keeping the N_A_ of CsPbI_3_ fixed on (a) PV performance (b) J-V curves.

The doping concentration is another critical parameter that directly impacts numerous optoelectronic parameters, i.e. V_oc_, carrier recombination rate, and diffusion length etc. [43]. As a result, understanding the role of doping mechanisms in the design of efficient solar cells is of immense importance. Initially, the acceptor density (N_A_) of MAPbI_3_ is varied from 10^14^ to 10^22^ cm^−3^ keeping the N_A_ of CsPbI_3_ fixed at 10^14^ cm^−3^. Figure 11 (a) and (b) depict the variation in N_A_ of MAPbI_3_ absorber layer on V_oc_, J_sc_, FF, and η and J-V curves of PSC. From the Figure, it is observed that, at low acceptor density, i.e., 1× 10^13^ cm^−3^ to 1× 10^15^ cm^−3^, there is a small amount of variation in V_oc_, J_sc_, FF, and PCE. But as the value of N_A_ increases greater than 10^16^ cm^−3^, we observe a sharp increase in V_oc_, FF, and PCE. The optimal value of PCE = 28.55%, V_oc_ = 1.23V, J_sc_ = 26.74 mA/cm^2^ and FF = 86.69% is obtained at N_A_ = 10^22^ cm^−3^. The observed rise in V_oc_ can be explained using the following equations:

(8) $$I_{\text{o}}=qn_{i}^{2}\left(\frac{D_{n}}{L_{n}N_{A}}+\frac{D_{p}}{L_{p}N_{D}}\right)$$

(9) $$V_{oc}=\frac{kT}{q}\text{ln}\left(\frac{I_{L}}{I_{\text{o}}}+1\right)$$

As it can be seen from the above equation that the rise in N_A_ would drop the saturation current I_o_, which will subsequently decrease the value of V_oc_.

**Figure 12:**
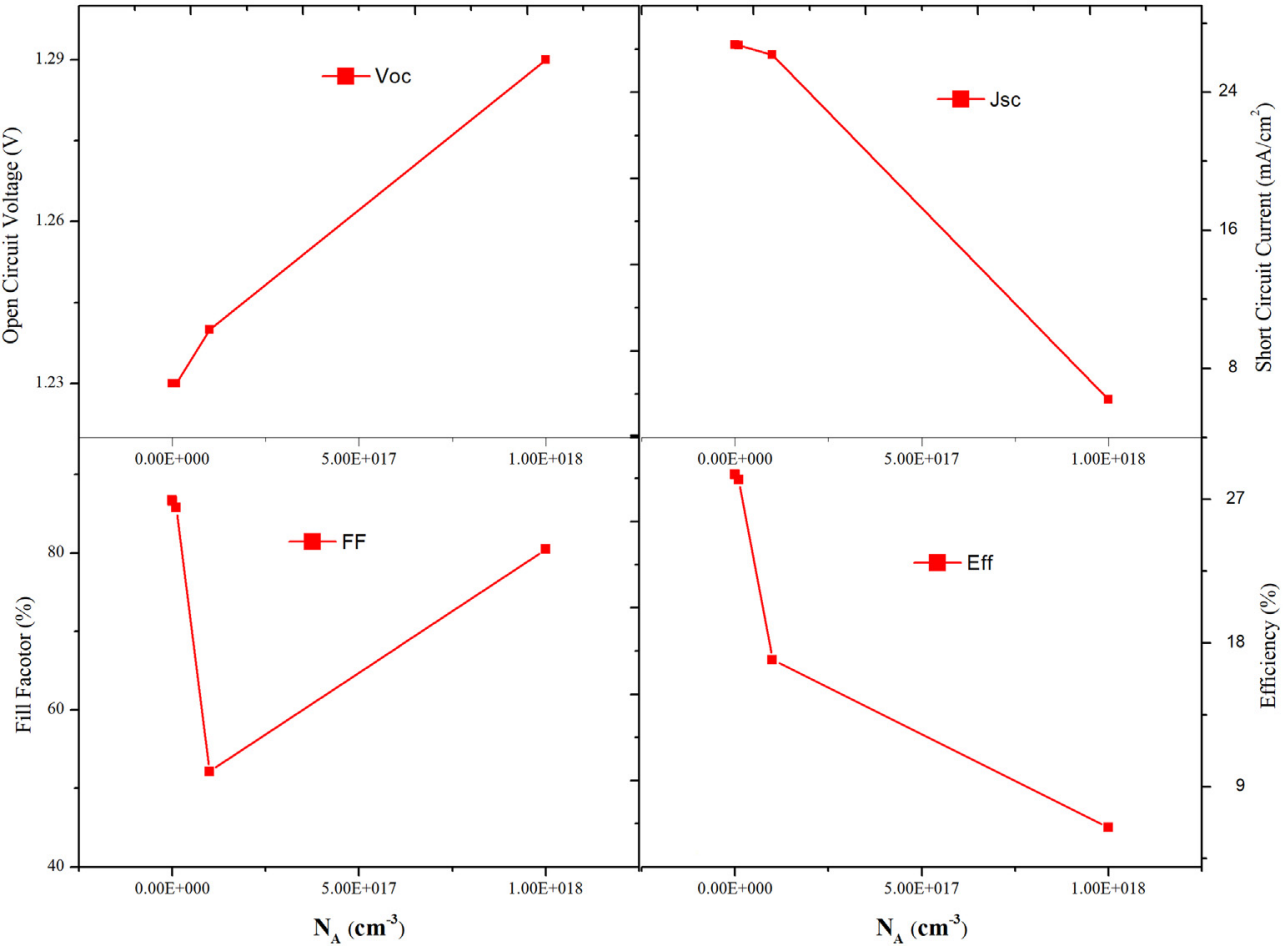
Effect of N_A_ variation in CsPbI_3,_ keeping the N_A_ of MAPbI_3_ constant on PV performance.

**Figure 13:**
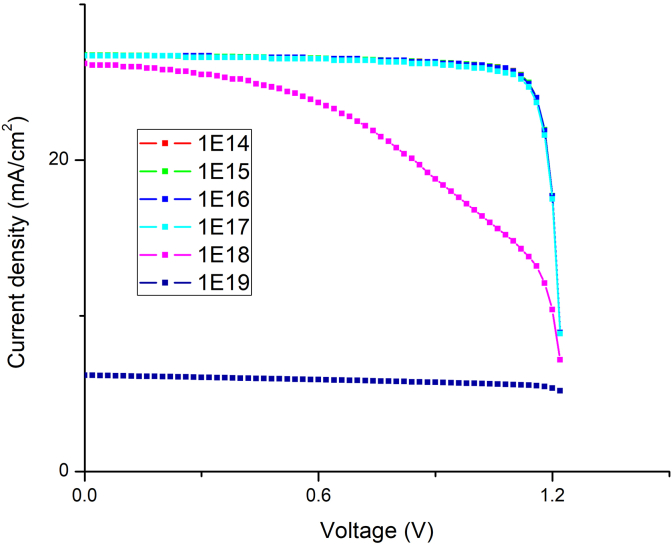
N_A_ variation in CsPbI_3_ J-V curves.

Similarly, the acceptor density of CsPbI_3_ was varied from 1× 10^13^ to 1×10^18^cm^−3,^ keeping acceptor concentration of MAPbI_3_ fixed at 1× 10^22^cm^−3^, and its effect on V_oc_, J_sc_, FF, and η was studied. Figures 12 and 13 shows the PV parameters and J-V curves with different N_A,_ respectively. Under low acceptor density, the J-V curves are nearly identical, as observed in Figure 13. When the N_A_ is more than 10^16^cm^−3^, the J_SC_ falls, and the V_OC_ rises. Eqs. (8) and (9) describe how the V_oc_ is improved, and the J_SC_ drops when N_A_ rises, there will be an optimal N_A_ that maximizes device efficiency, when N_A_ is 1×10^15^ cm^−3^, as shown in Figure 13, the highest PCE = 28.55% is achieved. Thus, for optimal performance, the value of N_A_ is 1×10^22^ cm^−3^ for MAPbI_3_ and 1×10^15^ cm^−3^ for the CsPbI_3_ absorber layer, respectively. Therefore, the above results show that in MAPbI_3_/CsPbI_3_ perovskite-perovskite bilayer PSC, the narrow bandgap material is more sensitive to the doping concentration than the wide bandgap material. Various techniques for passivating the traps have been investigated in the literature to improve crystallinity and grain size, chemicals such as metal ionic doping (K, Na, Cs), Lewis acid/base adduct, long-chain polymers, ammonium salts etc. [44, 45, 46].

### 3.6 Impact on work function of front contact

**Figure 14:**
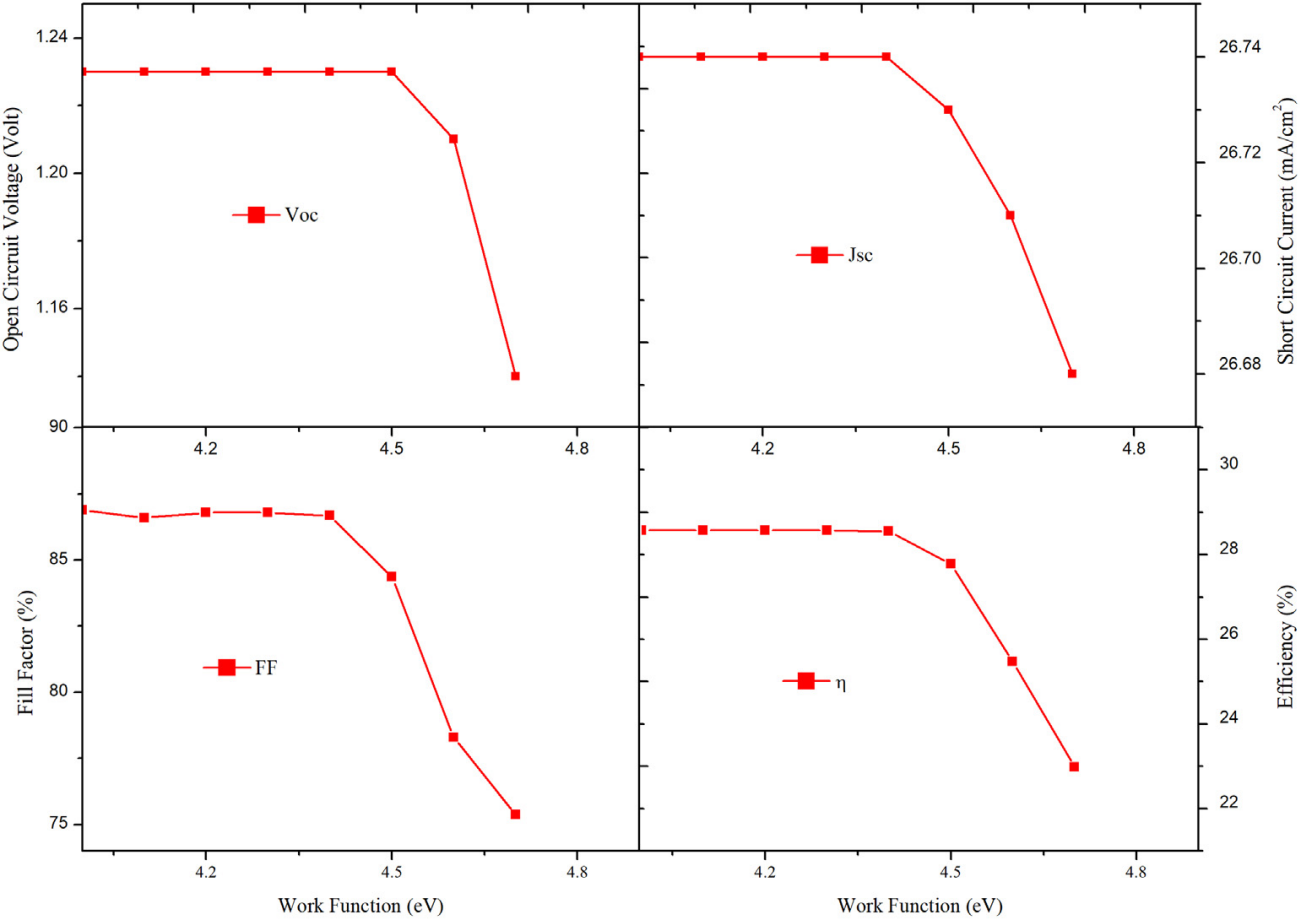
Effect of front contact variation on PV performance.

**Figure 15:**
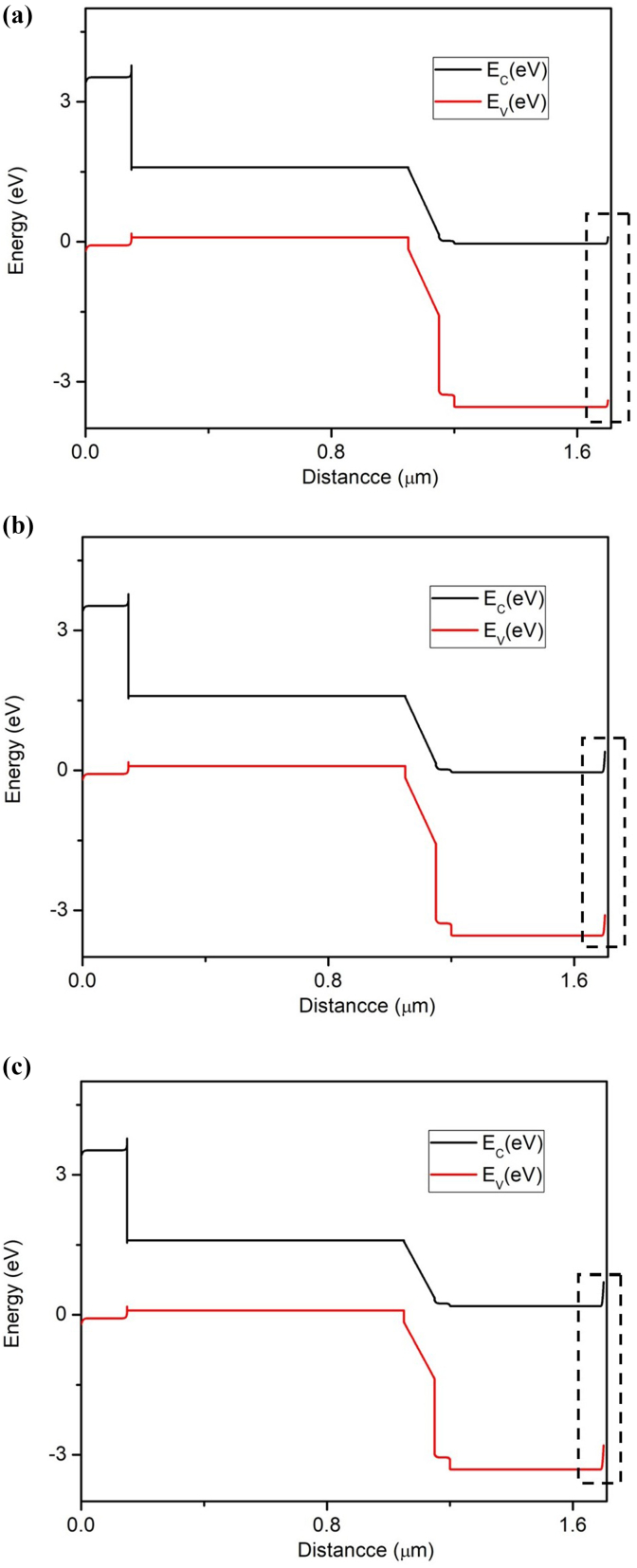
(a) 4.1 eV band diagram (b) 4.4eVband diagram (c) 4.7eV band diagram.

To assure appropriate collection of electrons from FTO (front contact), an ohmic contact should be established. The work function (φ) of the front contact was increased from 4.1eV to 4.7eV to explore its influence on V_oc_, J_sc_, FF, and η. Figure 14 depicts the effect of φ of front electrode on the performance of perovskite-perovskite bilayer PSC . As observed from Figure 15 that the PV performance remained the same when work function was increased up to 4.4eV only; as it was increased further, the device performance rapidly dropped. The reason behind the fall in the performance of PSC on increasing the work function of front contact is illustrated in Figure 15. As shown in Figure 15 (a), there is a minimal barrier when work function is 4.1eV; however, as it was increased, the barrier increased, as shown in Figure 15 (b) and 15(c), degrading the device's performance. The V_OC_, FF, and PCE started to degrade and reached 1.14 V, 75.37%, and 22.97%, from 1.23V, 86.69%, and 28.58%, respectively, because a high value of work function was creating a barrier for the flow of electrons at the interface of ETL and front contact. The barrier would rise as it was increased, lowering PV performance, as evident from Figure (15). Based on our simulations, we can deduce φ should be less than or equal to 4.4eV when choosing a material for the front electrode.

### 3.7 Effect of interface defect density

**Figure 16:**
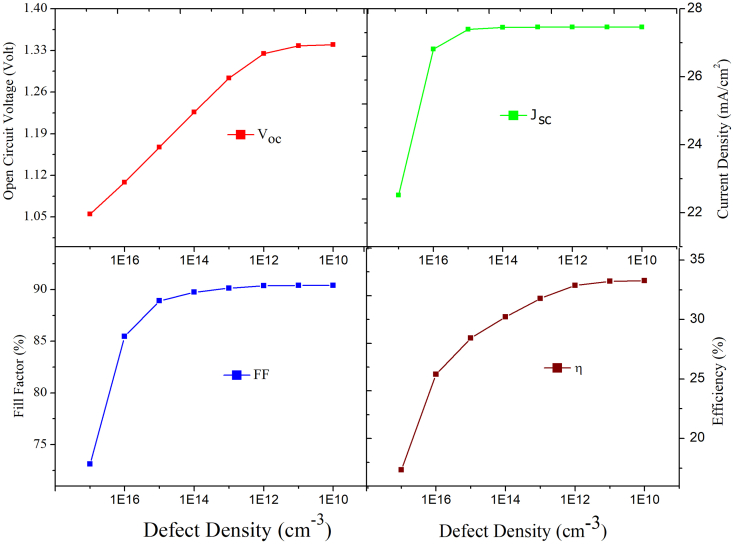
Effect of N_t_ interface at CsPbI_3,_/MAPbI_3_ interface on PV performance.

**Figure 17:**
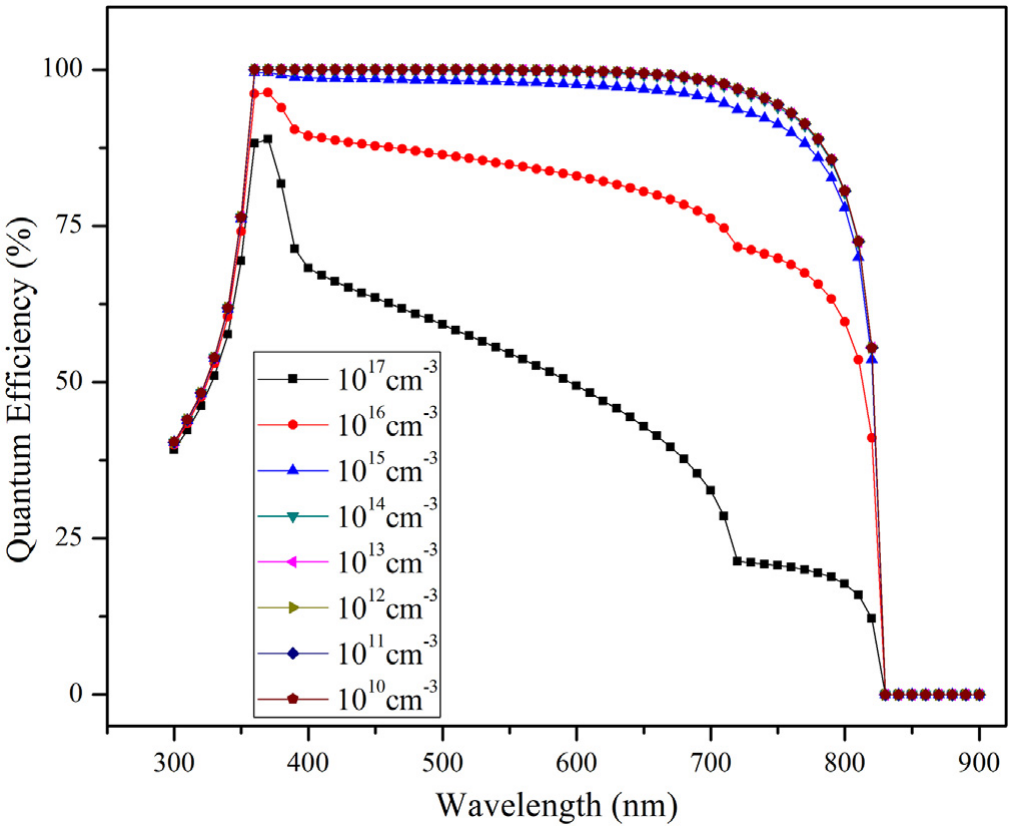
Quantum efficiency with variable interface defect density at CsPbI_3,_/MAPbI_3_ interface.

Interface recombination also plays a very important role in determining overall performance of the PSCs [47]. In this work the interface defect density (N_interface_) at the junction of the two-absorber layer is varied between 10^17^cm^−3^ to 10^10^cm^−3^ keeping other parameters fixed to examine its effect on V_oc_, J_sc_, FF and η. The PCE decreases from 33% to 7.48% while V_oc_ reduces from 1.34V to 1.02V, J_sc_ decreases from 27.45 mA/cm^2^ to 12.60 mA/cm^2^ and FF decreases from 90.41% to 71.94%. It was observed that the PV parameters changed drastically when the values of N_interface_ are varied between 10^17^cm^−3^ to 10^13^cm^−3^ while PV parameters are almost constant between 10^13^cm^−3^ to 10^10^cm^−3^ as observed from Figure (16). The drastic decline in device performance is due to interface defects which create additional trap states which serves as recombination centers for the charge carriers as N_interface_ increases. There is greater decrease in J_sc_ than V_oc_ on increase in defect density. This is due to interfacial recombination dominates charge-carrier density as N_interface_ increases. The sudden decrease in J_sc_ from 22 mA/cm^2^ to 12 mAcm^2^ on increase in N_interface_ is explained by quantum efficiency curve shown in Figure (17) where QE changes rapidly once (N_interface_) is greater than 10^17^cm^−3^. Thus, simulation reveals that interface engineering is necessary to have efficient solar cells. Interface engineering has been shown to be useful in minimizing power loss and recombination at various interfaces of PSCs [48]. Zhang successfully used ionic liquids as a modification layer which inhibit non-radiative recombination of charge-carriers at absorber layer, reduces energy level mismatch between perovskite film and carbon electrode. Thus ionic liquid passivates the surface defect of the perovskite absorber layer producing highly efficient and stable PSC [49]. As a result, we feel that a better knowledge of the working mechanism of PSCs can help improve device performance.

## 4 Conclusion

In this work, we have tried to optimize the efficiency of perovskite-perovskite bilayer PSC using the SCAPS-1D simulation tool. We have estimated the efficiency using different organic-inorganic materials, and among them, the best material and optimal thickness of the material is studied. The optimal perovskite-perovskite bilayer PSC shows 33.54% efficiency. Initially, single-junction CsPbI_3_ and MAPbI_3_ PSC were simulated and validated experimentally. The study was further continued for numerical simulation and analysis of perovskite-perovskite bilayer PSC with a MAPbI_3_/CsPbI_3_ heterojunction as the absorber layer. Then we have optimised for ETL material and HTL material. Furthermore, thickness, N_t_ and N_A_ of both of the absorber layers CsPbI_3_ and MAPbI_3_ were optimised.

The study shows that ZnO and CuSCN were the best ETL material and HTL material, respectively, because of better alignment of absorber layer valence band with HUMO and LUMO of ZnO and CuSCN, respectively. The proposed perovskite-perovskite bilayer PSC shows the highest efficiency at 100nm thickness for the CsPbI_3_ absorber layer while it is 900nm for the MAPbI_3_ absorber layer. Moreover, the perovskite-perovskite bilayer PSC shows the best performance at defect density of 1×10^13^ cm^−3^ and optimal performance in the range of 1×10^13^ cm^−3^ to 1×10^15^ cm^−3^ for both of the absorber layers. It was observed that the moderate doping concentration in the wide bandgap absorber layer and high doping concentration in the narrow bandgap absorber layer produce high efficiency.

The device shows optimized performance at a doping concentration of 1×10^15^cm^−3^ for CsPbI_3_ and 1×10^22^cm^−3^ for the MAPbI_3_ absorber layer, respectively. The proposed bilayer PSC shows the best PV performance at 4.1eV front electrode work function and optimal performance in the work function range 4.1eV–4.4 eV. Furthermore, PSC is optimized for defects at the interface of the two-absorber layer in the range 1×10^17^cm^−3^ to 1×10^10^cm^−3^ and the simulation results demonstrate that for optimal performance the interface defect density should be less than 1×10^13^cm^−3^. The PCE has increased from 20.39% before optimization to 33.54% after optimization in bilayer PSC, as shown in Table 8. The simulation results also demonstrate that bilayer PSC shows better performance than the single-junction PSC, i.e., either the CsPbI_3_ based PSC or the MAPbI_3_ based PSC. Thus perovskite-perovskite bilayer PSC gives higher efficiency than the single-junction PSC.

**Table 8:** PV performance before and after optimisation.

Bilayer PSC Parameters	Before optimisation	After optimisation
V_oc_ (volts)	0.93	1.34
J_sc_ (mA/cm^2^)	27.38	27.45
FF (%)	79.21	90.49
η (%)	20.39	33.54

## Declarations

### Author contribution statement

Sidra Khatoon: Conceived and designed the experiments; Performed the experiments; Analyzed and interpreted the data; Wrote the paper.

Satish Kumar Yadav: Analyzed and interpreted the data; Wrote the paper.

Jyotsna Singh: Contributed reagents, materials, analysis tools or data; Wrote the paper.

Rajendra Bahadur Singh: Contributed reagents, materials, analysis tools or data.

### Funding statement

This research did not receive any specific grant from funding agencies in the public, commercial, or not-for-profit sectors.

### Data availability statement

Data will be made available on request.

### Declaration of interests statement

The authors declare no conflict of interest.

### Additional information

No additional information is available for this paper.
